# From Thought to Action: How the Interplay Between Neuroscience and Phenomenology Changed Our Understanding of Obsessive-Compulsive Disorder

**DOI:** 10.3389/fpsyg.2015.01798

**Published:** 2015-11-23

**Authors:** J. Bernardo Barahona-Corrêa, Marta Camacho, Pedro Castro-Rodrigues, Rui Costa, Albino J. Oliveira-Maia

**Affiliations:** ^1^Department of Psychiatry and Mental Health, Faculdade de Ciências Médicas, Nova Medical School, Lisbon, Portugal; ^2^Department of Psychiatry and Mental Health, Centro Hospitalar de Lisboa Ocidental, Lisbon, Portugal; ^3^Champalimaud Clinical Centre, Champalimaud Centre for the Unknown, Lisbon, Portugal; ^4^Centro de Apoio ao Desenvolvimento Infantil, Cascais, Portugal; ^5^Centro Hospitalar Psiquiátrico de Lisboa, Lisbon, Portugal; ^6^Champalimaud Research, Champalimaud Centre for the Unknown, Lisbon, Portugal

**Keywords:** habitual behavior, goal-directed behavior, orbitofrontal cortex, uncertainty, action value

## Abstract

The understanding of obsessive-compulsive disorder (OCD) has evolved with the knowledge of behavior, the brain, and their relationship. Modern views of OCD as a neuropsychiatric disorder originated from early lesion studies, with more recent models incorporating detailed neuropsychological findings, such as perseveration in set-shifting tasks, and findings of altered brain structure and function, namely of orbitofrontal corticostriatal circuits and their limbic connections. Interestingly, as neurobiological models of OCD evolved from cortical and cognitive to sub-cortical and behavioral, the focus of OCD phenomenology also moved from thought control and contents to new concepts rooted in animal models of action control. Most recently, the proposed analogy between habitual action control and compulsive behavior has led to the hypothesis that individuals suffering from OCD may be predisposed to rely excessively on habitual rather than on goal-directed behavioral strategies. Alternatively, compulsions have been proposed to result either from hyper-valuation of certain actions and/or their outcomes, or from excessive uncertainty in the monitoring of action performance, both leading to perseveration in prepotent actions such as washing or checking. In short, the last decades have witnessed a formidable renovation in the pathophysiology, phenomenology, and even semantics, of OCD. Nevertheless, such progress is challenged by several caveats, not least psychopathological oversimplification and overgeneralization of animal to human extrapolations. Here we present an historical overview of the understanding of OCD, highlighting converging studies and trends in neuroscience, psychiatry and neuropsychology, and how they influenced current perspectives on the nosology and phenomenology of this disorder.

## Introduction

Obsessive-compulsive Disorder (OCD) is known to Western medicine at least since the Middle Ages (Berrios, 1996). The first historical accounts of OCD have been traced back to the 16th century, when people who suffered from repetitive blasphemous thoughts were believed to be possessed by demonic spirits (Muchembled, 2003). Phenomenologically, obsessions or obsessive thoughts have been considered to constitute the core of OCD. The term obsession derives from the Latin word *obsidere*, meaning to be possessed, occupied or preoccupied by something (Denys, 2011). It describes the occurrence of formal elements of thought (ideas, images, fears, doubts, ruminations) in a recurrent and persistent manner. These thoughts impose themselves on the individual, who experiences them as intrusive and anxiogenic, and incompatible with him or herself and/or his or her view of the world. In most cases, obsessive thoughts are accompanied by repetitive stereotyped behaviors, i.e., compulsions, from the Latin term *compellere*, meaning to be forced to something (Denys, 2011). Compulsions frequently assume a ritual-like nature and may either consist of motor acts (e.g., washing rituals) or purely mental acts (e.g., counting or praying; Burchard, 1980). Similarly to obsessions, compulsions are not in themselves pleasurable or gratifying to the patient. In most cases they are performed in order to reduce the anxiety evoked by obsessions, and are recognized by the subject as disproportionate or unrealistically related to the harm they are intended to avoid (Hollander et al., 2008).

Currently it is estimated that, in the USA, 2.3% of the adult population suffer from this condition (American Psychiatric Association, 2000). Moreover, there is evidence suggesting under-diagnosis (Fullana et al., 2009) and that isolated obsessive-compulsive symptoms (OCS) are extremely frequent in the general population (Grabe et al., 2001). OCD tends to present a chronic course and is frequently comorbid with mood disorders, eating behavior disorders and substance abuse (Torres et al., 2006). The World Health Organization (Ayuso-mateos, 2000), in an analysis of the indirect costs of the disorder (e.g., inability to work, impact on the family, early retirement) placed OCD in the 11th position of diseases with the greatest impact of non-fatal disease burden, at a level similar to schizophrenia in terms of years lost to disability.

From the early times of modern psychiatry, OCD has exerted a particular fascination on clinicians and researchers alike. Over more than a century of clinical and neurobiological research, OCD moved from being considered a typical neurosis (Black, 1974) to being the prototypical neuropsychiatric disorder, or at least, the most accomplished example of a mental disorder with clear underlying biological correlates. In the process, our understanding, and even our wording, of the disorder’s manifestations changed radically (Berrios, 1989). Here we briefly review the current state of the art with regard to the neurobiology of OCD, and how neurobiological models have opened new perspectives on the phenomenology of this disorder.

## Classification of OCD

The first early modern descriptions of OCD already acknowledged the composite nature of this nosological entity. Indeed, in his Études cliniques sur les maladies mentales et nerveuses, Jules Falret was the first to comment on a clinical distinction between *folie du doute* (doubting madness) and *délire du toucher* (touching madness; Falret, 1890). More recently, factor analytical studies of OCS have consistently confirmed this notion and defined 4 to 5 clusters of OCS: 1. symmetry and “just right” obsessions with counting, ordering or repeating compulsions; 2. contamination obsessions with washing and cleaning compulsions; 3. hoarding compulsions; 4. aggressive obsessions with superstitious and checking compulsions; 5. sexual and religious obsessions (in four-factor models categories 4 and 5 are grouped together; Mataix-cols et al., 2005; Bloch et al., 2008; Landeros-Weisenberger et al., 2010). In a prospective study, it was shown that, even though specific obsessions may vary in individual patients over time, they generally tend to remain within the same broad symptomatic category (e.g., obsessions about body fluids evolve to excessive concern with contagious illnesses; Mataix-cols et al., 2005). Importantly, both structural and functional imaging studies support the hypothesis that particular neurobiological substrates underlie these different phenomenological dimensions of OCD (Menzies et al., 2008). Moreover, there is consistent evidence that distinct symptom dimensions may respond differently to specific treatments (Mataix-cols et al., 2005). In any case, such attempts to divide OCD into distinct entities have had a limited impact on the classification of the disorder.

Since its original inclusion in the first edition of the Diagnostic and Statistical Manual of Mental Disorders (DSM-I; American Psychiatric Association, 1952), that classified OCD as a neurotic disorder, OCD has remained a single discrete nosological entity, included in subsequent editions of the manual within the broader category of the anxiety disorders. It was only in the very recent 5th edition of DSM that the nosological heterogeneity of OCD has been acknowledged for the first time, with hoarding being considered as a distinct, albeit related, disorder. Moreover, the two disorders were removed from the Anxiety Disorders category and placed in a novel category—Obsessive Compulsive and Related Disorders—comprising OCD and hoarding, as well as body dysmorphic disorder, trichotillomania and compulsive skin-picking. The decision to create this new nosological category was based on the increasing evidence that these disorders are related in terms of phenomenology, comorbidity, neural substrates, and treatment response (American Psychiatric Association, 2013). The new DSM category additionally reflects the notion that OCD constitutes one of the extremes of an obsessive-impulsive spectrum of disorders, that hold in common a failure of inhibitory behavioral control (Hollander et al., 2008). Psychopathology at the obsessive end of this spectrum mainly revolves around harm avoidance, with reward-seeking predominating at the opposite end of the spectrum and materializing in disorders such as pathological gambling or certain sexual paraphilia’s. Although this view of an obsessive-impulsive spectrum has been challenged by accumulating evidence from both human and animal-based research showing that obsessiveness and impulsiveness are actually orthogonally related dimensions, the construct of an obsessive-impulsive group of syndromes, with psychopathology combining varying intensities of reward-seeking (impulsiveness) and harm-avoidance (obsessiveness), continues to be supported by many authors (Fineberg et al., 2010).

## Neurobiology of OCD

### Neuroanatomy of OCD

The concept of OCD as a neuropsychiatric disorder of behavior emerged initially in the first quarter of the 20th century from observations that OCD frequently developed as a complication of encephalitis lethargica, and that subtle neurologic signs, similar to those present in recovered encephalitis patients, occur frequently in primary OCD (Schilder, 1938). Constantin von Economo was the first to describe, in 1931, the association between OCD and post-encephalitic Parkinsonism, and was also the first to suggest a causal link between basal ganglia lesions and secondary OCD (Schilder, 1938; Swedo and Snider, 2004).

Globally, the findings of structural and functional neuroimaging studies in OCD seem to converge on the orbitofrontal corticostriatal circuit and its limbic connections, mainly the cingulate gyrus and the temporal amygdala. The most consistent and replicated evidence from structural studies is of a reduction in gray matter and white matter volume of the orbitofrontal cortex (OFC), medial frontal gyrus, and anterior cingulate cortex (ACC), and increased volume of the ventral putamen (Pena-Garijo et al., 2010; De Wit et al., 2014). The most replicated findings of functional imaging studies tend to concentrate on these very same structures: most reports describe an increase in metabolic activity or in blood-oxygen-level dependent (BOLD) signal intensity in the orbitofrontal corticostriatal circuit, and a positive correlation between OCS severity and activity in that circuit (Westenberg et al., 2007; Pena-Garijo et al., 2010; Freyer et al., 2011). Furthermore, such activity is sensitive both to symptom provocation and to successful pharmacological or psychotherapeutic treatment. One study, combining meta-analysis of voxel-based morphometry studies and of functional neuroimaging studies with symptom provocation, demonstrated that, in OCD patients, the lateral OFC is the only brain region where structural findings coincide with functional findings (Rotge et al., 2010). It is, however, impossible to ignore the consistency of findings concerning other significant brain regions, namely the parietal cortex, the dorsolateral prefrontal cortex (dlPFC), the ventrolateral prefrontal cortex, the cerebellum and the caudate (Westenberg et al., 2007; Guehl et al., 2008; Menzies et al., 2008; De Wit et al., 2014).

### Neurochemistry of OCD

#### Serotonin

Most of the evidence supporting a role for serotonin in the pathophysiology of OCD is based on the established therapeutic effects of serotonin reuptake inhibitors and the inefficacy of antidepressants with no effect on serotonin transmission or metabolism (Baumgarten and Grozdanovic, 1998; Westenberg et al., 2007; Goddard et al., 2008). Positron emission tomography (PET) and single positron emission computed tomography (SPECT) studies have shown a decreased availability of postsynaptic 5-HT2A receptors in the dlPFC, OFC and temporo-parietal cortices of OCD patients, as well as a reduced density of serotonin reuptake transporters in the striatum (Hesse et al., 2005; Westenberg et al., 2007; Perani et al., 2008). Both of these changes correlate with OCS severity. In spite of its popularity, the evidence base supporting the serotonergic model of OCD is mostly indirect and conceptually fragile. For many authors the available evidence suggests a role for serotonin not so much in the etiology of OCD as in the mechanism of action of the drugs used in its treatment (Baumgarten and Grozdanovic, 1998; Westenberg et al., 2007). In fact, only approximately half of OCD patients show any significant improvement when treated with serotonin reuptake inhibitors, a lower response rate than the one obtained in depression using the same agents. Moreover, in OCD patients, tryptophan depletion has no discernible effect on symptoms (Baumgarten and Grozdanovic, 1998; Westenberg et al., 2007).

In animal studies, evidence for a role of serotonin in compulsive-like behaviors has also been presented. In rats developing compulsive-like behaviors after OFC lesions, an increase of serotonin transporter density was found in the striatum (Joel et al., 2005). Stereotyped behavior can also be obtained pharmacologically, in rodents, by administration of 5-HT-2A antagonists (Fineberg et al., 2010), and decreased 5-HT-2A receptor activation decisively impairs reversal learning by increasing the number of perseverative responses to previously reinforced stimuli (Boulougouris et al., 2008). Furthermore, systemic administration of 5-HT-2A antagonists, or local infusion into the nucleus accumbens or medial prefrontal cortex (mPFC), *reduces* impulsivity in animal models, which might partially explain the limited success of “serotonergic” treatments in OCD (Boulougouris et al., 2008). Interestingly, while 5-HT-2C knock-out mice were found to develop compulsive chewing of a non-nutritive substance in one study (Chou-Green et al., 2003), treatment with the non-specific 5-HT-2C agonist m-chlorophenylpiperazine acutely induces OCS in humans and compulsive-like behaviors in animals (Tsaltas et al., 2005; Fineberg et al., 2010). 5-HT-2C antagonists, on the other hand, decrease perseverative and total number of trials in reversal learning tasks, and exacerbate premature responses (i.e., impulsivity), when administered systemically or when infused directly into the nucleus accumbens (Winstanley et al., 2004; Boulougouris et al., 2008).

#### Dopamine

One of the main arguments for a role of dopamine in the pathophysiology of OCD is the beneficial effect of antipsychotic medication in many cases that are resistant to SSRIs, particularly when tics are present (Miguel et al., 2001; Perani et al., 2008). Furthermore, OCS are frequent in movement disorders associated to hyperdopaminergic dysfunction, such as Sydenhams’s chorea or Huntington’s disease. In Parkinson’s disease, OCD and other behaviors of the impulsive-compulsive spectrum have been described, mostly, though not exclusively, in patients treated with high doses of L-dopa (Evans et al., 2004; Koo et al., 2010). Finally, there has been extensive demonstration of both the induction of *de novo* stereotyped compulsive-like behaviors and the aggravation of pre-existing OCS by drugs that stimulate dopamine receptors directly (e.g., bromocriptine, apomorphine) or indirectly (e.g., amphetamines, cocaine, methylphenidate; Denys et al., 2003; Perani et al., 2008; Koo et al., 2010). Treatment with bupropion, a dopamine reuptake inhibitor, has also been shown to aggravate OCD symptoms in a small trial (Vulink et al., 2009).

Similarly to what has been described for serotonin, direct evidence of a role for dopamine in the neurobiology of OCD also derives from PET and SPECT studies, which have revealed an increased density of dopamine transporter (DAT; Landeros-Weisenberger et al., 2010) and a reduction of D2 receptor availability in the basal ganglia (Denys et al., 2003; Kim et al., 2003). In some studies these changes were reverted after treatment with a selective serotonin reuptake inhibitor (SSRI; Kim et al., 2003; Moresco et al., 2007). One study reported a lower availability of D1 receptors in the striatum of OCD patients when compared to healthy subjects (Olver et al., 2009). In line with these findings, DAT knockout mice, which present synaptic dopamine levels 70% or more above normal, have stereotyped grooming behaviors, especially when exposed to unfamiliar environments (Wang et al., 2012). Furthermore, an often-cited animal model for OCD consists of repeated subcutaneous injections of the D2-agonist quinpirole, which leads to stereotyped, compulsive-like behavior in rats. This phenotype reverts with clomipramine treatment and subthalamic nucleus (STN) inactivation through deep brain stimulation (DBS; Winter et al., 2008; Klavir et al., 2009).

A potential role for dopamine in OCD has been frequently interpreted in the context of evidence for the participation of this neurotransmitter in reinforcement learning (Pizzagalli et al., 2008; Wise, 2009), including negative reinforcement learning, i.e., the performance of a behavior to prevent anticipated adverse consequences (Pessiglione et al., 2006). In OCD patients, a hypothetical excess of dopaminergic input into the ventral striatum could lead to excessive aversion avoidance behaviors, as well as to failures in reversal learning (Wunderlich et al., 2011). This prediction was recently partially confirmed in a study showing that, in a shock avoidance task, OCD patients will sustain avoidant responses beyond the interruption of shock delivery (Gillan et al., 2014a). Additionally, mesocorticolimbic dopaminergic hyperactivity could enhance the reinforcement of anxiety-reducing stereotyped actions, the performance of which would thus become rewarding and confer to compulsions properties akin to those of other “behavioral addictions” (Holden, 2001; Aouizerate et al., 2004; Wise, 2009). However, there is also evidence that ventral striatum activation during reward anticipation is blunted in OCD, which may be inconsistent with this hypothesis (Figee et al., 2011).

While there is extensive evidence pointing toward increased synaptic concentration of dopamine in the striatum of OCD patients, there is also evidence suggesting the reverse (Sesia et al., 2013). One SPECT study found reduced striatal DAT density in drug-naïve OCD patients when compared to healthy controls (Hesse et al., 2005). In another study midbrain DAT density was increased after citalopram treatment (Pogarell et al., 2005). Moreover, the risk of developing OCD appears to be increased in chronic cocaine abusers, who have down-regulated dopamine receptors and dopamine function (Crum and Anthony, 1993). There are also isolated reports of OCD cases where treatment with amphetamine, methylphenidate or bromocriptine led to an improvement of OCS—an intriguing observation given the often described aggravation of symptoms in OCD patients exposed to these substances (Denys et al., 2003; Perani et al., 2008; Koo et al., 2010). Furthermore, most atypical antipsychotics, and even haloperidol, can, in some patients, cause significant OCS (Lykouras et al., 2003). In summary, there is a definite role for dopamine, and possibly dopamine-signaled reward processing, in the pathophysiology of OCD, but the more exact characteristics of this role are still unclear.

#### Glutamatergic and GABAergic Neurotransmission in Corticostriatal Circuits

Recent findings suggest the involvement of other neurotransmitter systems, beyond serotonin and dopamine (Kariuki-Nyuthe et al., 2014). The unequivocal evidence that corticostriatal pathways are hyperactive in OCD has led to research into changes of glutamatergic neurotransmission, specifically at corticostriatal synapses. There is some evidence that, in OCD patients, the concentration of glutamate in the cerebrospinal fluid is increased (Ting and Feng, 2008), and magnetic resonance spectroscopy studies have found evidence of increased glutamate levels in the OFC, ACC, and striatum (see Naaijen for a review; Naaijen et al., 2015). In some studies, striatal glutamate levels have been found to correlate with OCS severity (Starck et al., 2008), and to decrease after pharmacological treatment or cognitive-behavioral (Rosenberg et al., 2000; O’Neill et al., 2013). Furthermore, some genes of interest in OCD research, identified in animal models of the disorder, are related to glutamatergic receptors (such as the GRIN2B and SLC1A1 genes) or to corticostriatal glutamatergic synapses (SAPAP3 gene; Wan et al., 2014). Consequently, in the last years, several pharmacologic agents that modulate glutamatergic transmission have been tested in OCD, such as memantine, topiramate, and riluzol (that reduces the synaptic release of glutamate). While the results are, in general, promising, these studies are still rare, mostly unreplicated, and based on small samples (Ting and Feng, 2008).

Gamma-aminobutyric acid (GABA), the main inhibitory neurotransmitter in the central nervous system, has been much less studied in OCD. Magnetic resonance spectroscopy studies found decreased levels of GABA in prefrontal cortical areas, including the ACC (Simpson et al., 2012), with one study reporting that an acute increase in mPFC GABA levels coincides with OCS relief after successful ketamine treatment (Rodriguez et al., 2015). Moreover, studies using transcranial magnetic stimulation found shortened cortical silent periods and increased intracortical facilitation in the left motor cortex of OCD patients—both of which are considered measures of GABA_*B*_ receptor mediated neuronal inhibition (Richter et al., 2012). Taken together, these observations have been interpreted as reflecting reduced activity or number of cortical GABAergic interneurons which could, both directly and indirectly (through ACC to OFC projections), lead to abnormal striatal activation (Simpson et al., 2012). In line with this hypothesis, an association has been found between polymorphisms in the GABA_*B*_ receptor 1 gene and OCD (Zai et al., 2005). Finally, studies in primates have shown that injecting a GABA_*A*_ receptor antagonist (bicuculline) in specific areas of the ventral striatum leads to OCD-like behavior changes (Worbe et al., 2009).

### Genetic Studies in OCD

Environmental factors are relevant for the occurrence of OCD (Krebs et al., 2015). Maternal consumption of alcohol or caffeine during pregnancy, *hyperemesis gravidarum* or dystocic labor, adverse life events, including childhood physical or sexual abuse, were all found to increase the risk of developing this disorder (Cath et al., 2008). Nevertheless, it is not rare to uncover, in patients with OCD, a family history of the disorder, especially in cases with disease onset during childhood or adolescence, with co-morbid tics or with a predominance of symmetry/organization symptoms (Nicolini et al., 2009; Samuels, 2009). Thus, a possible role for genetic factors in OCD has been explored since at least 1929 (Lange, 1929). While many of the earlier studies had important methodological limitations (Pauls, 2010), more recent twin studies have shown that 67.5% of homozygous twins are concordant for OCD, with 31% concordance in dizygous twins (Aouizerate et al., 2004). The estimated contribution of genetic factors for the risk of developing OCD varies between 45 and 65% for child-onset disease, and 27 to 47% for adult-onset OCD (Nicolini et al., 2009), with genetic association studies pointing mostly to genes related to the serotonergic and dopaminergic systems (Frisch et al., 2000). Importantly, theoretical models derived from genetic segregation analysis suggest the simultaneous existence of mendelian (monogenic) and polygenic genetic transmission (Nicolini et al., 2009), with the description, in linkage studies, of major genetic loci in chromosomes 1, 3, 6, 7, 9, 10, 11, 14, and 15 (Samuels, 2009).

### Neural Circuit Models of OCD

From the currently available information, two models have been proposed regarding OCD pathophysiology: the “cortical” model, centered primarily on frontal cortex dysfunction, and the “subcortical” model, that attributes OCD to dysfunction of the basal ganglia and their cortical connections (Modell et al., 1989; Deckersbach et al., 2006). The proponents of both models tend to focus on the importance of neurodevelopmental factors, given the frequent onset during childhood or adolescence, the comorbidity with developmental disorders, namely autism spectrum disorders, and the presence of discreet neurological signs in patients suffering from OCD (Bradshaw, 2001).

The cortical model for the neurobiology of OCD essentially proposes that OCD results from an imbalance between hyperactive OFC and ACC and hypoactivity of the dlPFC. According to this model, hyperactivity of the OFC and ACC, which has been consistently demonstrated in OCD, elicits egodystonic behavioral commands and uncertainty error signals (Bradshaw, 2001; Westenberg et al., 2007). These error signals are resistant to feedback information from sensory and limbic areas leading to persistent ruminations of uncompleteness or doubt and behavioral perseveration with stereotyped responses such as verification and repetition, aiming to reduce uncertainty (Pena-Garijo et al., 2010). Activity in the nucleus accumbens and amygdala would be secondarily modulated through extensive reciprocal connections with the OFC and ACC, leading to the profound anxiety and apprehension that is characteristic of OCD (Westenberg et al., 2007). Recent data on resting-state corticostriatal connectivity in children with OCD are broadly consistent with this hypothesis: reduced connectivity between the dorsal striatum and ACC coexists with increased connectivity between both dorsal and ventral striatum and medial frontal cortical areas involved in emotional processing (Fitzgerald et al., 2011). The net result would be an inability to suppress pre-potent security concerns and error signals while simultaneously investing them with excessive emotional salience (Fitzgerald et al., 2011; Baioui et al., 2013). Also consistent with this ventral-dorsal imbalance cortical model, decreased connectivity between the caudate nucleus and the dlPFC has been shown in OCD, possibly contributing to the cognitive inflexibility that is described in OCD, and participating in the inability to suppress thoughts and behaviors that are maladapted to environmental circumstances (Westenberg et al., 2007; Harrison et al., 2009). The classical cortical model thus ascribes OCD to a primary prefrontal dysfunction, essentially consisting of an imbalance between decreased dlPFC activity and increased activity in the OFC and ACC (McGuire, 1995; Fuster, 1997; Aouizerate et al., 2004). A less cited variant of the cortical model of OCD considers that intrusive feelings of anxiety originate from primary hyperactivity of the temporal amygdala. Such hyperactivity would lead to a secondary supplementary inhibitory effort from the medial and orbital prefrontal cortex. This supplementary inhibitory effort would be compromised by mesolimbic dopaminergic hyperactivity, that has been shown to reduce cortical inhibitory control over the amygdala (Denys et al., 2003; Westenberg et al., 2007).

The subcortical model of OCD neurobiology, first suggested by Modell (Modell et al., 1989), is currently the most popular and most frequently cited of the two models. According to this author, OCD results from a failure of the ventral pallidum to inhibit the mediodorsal thalamic nucleus and its connections to the OFC. This pallidal dysfunction would, at least in part, result from an unbalance between the activity of the main ventral striatum midbrain afferents, namely excitatory dopaminergic pathways from the ventral tegmental area, and inhibitory serotonergic pathways from the raphe nuclei (Aouizerate et al., 2004; Westenberg et al., 2007). The functional consequence would be an “anomalous reverberation” of the orbitofrontal cortico-striato-thalamo-cortical circuit. From a neurochemical perspective, the subcortical models are consistent with the role traditionally ascribed to hypoactive ascending serotonergic pathways in the physiology of OCD and with current evidence supporting a role for dopamine in the neurobiology of OCD and other disorders of the obsessive-compulsive spectrum.

In the last two decades, the subcortical model has been reviewed and perfected to accommodate novel research findings. According to the update proposed by Baxter et al (Baxter, 1987, 1992; Valente et al., 2005), OCD would result from a disruption of the caudate’s “filter” function. The consequence would be a self-sustained, reverberant release of automatic programs, with a need for supplementary efforts to maintain adequate responses to relevant stimuli (Baxter, 1987, 1992). At the microstructural level, dysfunction of the caudate would mainly involve the striosomal compartment, which is predominantly implicated in the direct pathway of the basal ganglia. Striosomes are particularly numerous in the ventromedial striatum, where they receive afferents from the OFC and ACC (Eblen and Graybiel, 1995; Aouizerate et al., 2004). In the Baxter model, hyperactivity in limbic and orbitofrontal corticobasal circuits is compounded by hypoactivity of the dlPFC, which has been consistently described in functional neuroimaging studies of OCD (Aouizerate et al., 2004; Menzies et al., 2008; Nicolini et al., 2009). Projections from the dlPFC to neurons in the striatal matrix are proposed to influence the indirect pathway, thus participating in the interruption of automatic behaviors and the adaptive switch from one behavioral program to another (Baxter, 1987, 1992).

#### Cortico-subcortical Models

In 2005, Chamberlain updated Baxter’s model, centering OCD pathophysiology on the orbitofrontal corticostriatal circuit and its role in the acquisition and maintenance of stereotyped and automatic cognitive and behavioral patterns (Chamberlain et al., 2005; Menzies et al., 2008; Fineberg et al., 2010). The dysfunction of this network was proposed to explain the inability to suppress intrusive thoughts and automatic behaviors, as well as the deficits of psychomotor and cognitive inhibition that are abundantly described in OCD (i.e., the impulsive dimension of OCD phenomenology). These deficits of cognitive inhibition would in turn impair cognitive flexibility, that requires inhibition of prior cognitive processes, and working memory, that requires active suppression of distracting elements, both of which sustain the compulsive dimension of the OCD’s phenotype (Chamberlain et al., 2005). The greatest merit of this model is that it reassigns a decisive role to cortical dysfunction and top-down cortical control of frontostriatal circuits (Fineberg et al., 2010). It thus avoids the somewhat arbitrary dichotomy between cortical and subcortical models. Moreover, it is more in line with recent evidence that the direct and indirect pathway do not necessarily exert a competitive influence on action control or selection, but rather act in coordination for adaptive action control (Cui et al., 2013).

Central to all of the successive subcortical models of OCD lies the concept of subtle dysfunction of the ventral caudate and of its role in activating and maintaining behavioral programs that are adaptive to updated information, transmitted by associative cortical regions (Aouizerate et al., 2004; Westenberg et al., 2007). Support for a central role of the caudate in the physiology of OCD has been provided by the demonstration, *in vivo*, of an abnormally high rate of neuronal depolarization in the caudate of patients with severe OCD undergoing DBS (Guehl et al., 2008). Furthermore, modulation of orbitofrontal corticostriatal circuitry using optogenetics can induce or decrease obsessive-compulsive-like behaviors in mice (Burguière et al., 2013; Ahmari et al., 2014), and DBS of frontostriatal targets has been shown to ameliorate OCS in OCD patients (Figee et al., 2013). Indeed, trials of DBS for OCD have revealed an important role for the STN in this disorder. It has been shown that the cortico-STN pathway (i.e., the hyperdirect pathway) is required to interrupt ongoing automatic motor and behavioral programs, before they become overtly expressed (Wylie et al., 2010; Alegre et al., 2013; Anzak et al., 2013). Consistent with this, in a recent study of OCD patients submitted to DBS, OCD severity was related to neuronal activity in the associative-limbic subdivision of the STN, with shorter bursts and interburst intervals, as well as higher intraburst frequency, in OCD patients (Welter et al., 2011).

## The Evolving Neuropsychology of OCD

Despite the substantial body of literature published over the last 25 years on neuropsychological performance in OCD, studies have yielded inconsistent results, mainly attributable to methodology differences (Abramovitch et al., 2013). The cognitive deficits that are more consistently described in OCD patients are found in tasks of non-verbal memory, response inhibition, interference control, cognitive flexibility, and visuospatial working memory, with executive dysfunction being the hallmark of OCD neuropsychological profile (Kashyap et al., 2013; Abramovitch and Cooperman, 2015). Despite the evidence of impaired executive functioning in OCD, it is not consensual whether these deficits are trait-related (stable individual characteristics) or state-dependent (dependent on the presence of symptoms). In general, there seems to be more support for the former hypothesis, since there is no correlation between the intensity of cognitive changes and the severity of OCS. In a longitudinal study, where executive functions were tested in OCD patients both before and after symptomatic remission, executive function deficits were found to be stable over time and independent of symptom remission (Bannon et al., 2006). Furthermore, treatment of OCS does not lead to improvements in cognitive performance suggesting that, in OCD, cognitive deficits are not merely a side effect of the core psychiatric symptoms (Kuelz et al., 2004).

Taken together, most studies suggest that cognitive deficits in OCD reflect OFC and dlPFC dysfunction as well as, possibly, temporo-parietal dysfunction of the non-dominant hemisphere (Whiteside et al., 2004; Menzies et al., 2008). In functional brain-imaging studies comparing the pattern of cortical activation between OCD patients and healthy individuals, cognitive deficits are associated both with decreases and increases of cortical BOLD signal or glucose metabolism over the OFC, dlPFC, and temporo-parietal cortices (Balleine and O’Doherty, 2010; Banca et al., 2015). The reason for such incongruences is unclear, but it has been ascribed both to state-dependent differences in cortical activation (Page et al., 2009; Banca et al., 2015), and to discrepancies in the interpretation of results from different imaging techniques (Whiteside et al., 2004).

Several authors have also underlined the analogy between core obsessive-compulsive psychopathology and cognitive deficits in OCD patients. Specifically, the inability to repress compulsive behaviors is interpreted in the context of difficulties in performing response inhibition tasks, while the inability to interrupt obsessive thoughts is related to difficulties in performing cognitive flexibility tasks (Pena-Garijo et al., 2010). Others have further suggested that, more than a simple phenomenological analogy, a genuine correspondence exists between impulsivity and response inhibition deficits, and between compulsivity and deficits in cognitive and behavioral flexibility (Fineberg et al., 2010). Here, impulsivity is defined as a predisposition toward rapid, unplanned reactions to internal or external stimuli, with little regard for the adverse consequences of these actions. Compulsivity on the other hand is defined as a tendency to perform unpleasant repetitive actions in a stereotyped manner, in order to prevent perceived or anticipated negative consequences of not performing these actions. These behavioral dimensions are proposed to be sustained by two parallel corticostriatal circuits: a compulsive circuit, where compulsive behaviors are driven by the dorsal striatum and inhibited by the OFC; and an impulsive circuit where behavior is driven by the ventral striatum/nucleus accumbens shell, and inhibited by the ACC and ventromedial prefrontal cortex (vmPFC; Balleine and O’Doherty, 2010; Fineberg et al., 2010). In summary, the two core neuropsychological deficits described in OCD not only parallel the two main behavioral dimensions of this disorder—impulsivity and compulsivity—but also converge strikingly with the host of data pointing to structural and functional changes in the limbic and associative corticobasal circuits, and to deregulation of the two main midbrain monoaminergic ascending pathways (Aouizerate et al., 2004; Fineberg et al., 2010).

### OCD and Action Control

More recently, advances in our understanding of the neurobiology of action control, specifically the distinction between goal-directed and habitual action strategies, have opened new perspectives on the neurobiology of OCD. Goal-directed behaviors are those that are sensitive to revaluation of the rewarding outcome (e.g., feeding to satiation), and also to degradation or extinction of the contingency between performing the action and obtaining the outcome. Habitual behavior, on the other hand, persists even when the outcome is no longer rewarding or the action-outcome contingency is degraded or reversed. The phenomenological analogy between habitual action control and compulsive behavior has led to the hypothesis that OCD patients may be predisposed to rely excessively on habitual action control rather than on goal-directed behavioral strategies (Gillan et al., 2011; Robbins et al., 2012). Evidence that patients suffering from OCD present structural and functional changes in the striatum and mPFC, areas so frequently implicated in the control of transitions between action strategies (Tricomi et al., 2009; Balleine and O’Doherty, 2010), further strengthens this hypothesis. Recently, in a series of studies with OCD patients, using diverse decision making paradigms, Gillan and colleagues provided empirical support for the hypothesis that such patients are biased toward habitual action control patterns, in comparison with control subjects (Gillan et al., 2011, 2014a), and fail to rely on prospective comparisons of action-outcome alternatives to guide decision making (Gillan et al., 2014b). In an fMRI study with symptom-provocation in OCD patients, a pattern of activation/deactivation was evoked, suggesting an imbalance in circuits involved in the control of habitual and goal-directed action strategies (Banca et al., 2015). In fact, the authors describe deactivation of caudate-prefrontal circuits simultaneous to hyperactivation of putamen and STN regions, which was not found in control conditions, or in healthy subjects. Importantly, in additional connectivity analyses, the vmPFC and OFC were found to be a critical node in the circuits involved in symptom provocation (Banca et al., 2015).

However, the parallel between habitual action patterns and the elaborately ritualized, egodystonic behaviors that plague OCD patients may be an over-simplification. Importantly, the approaches used to date to study action control patterns in OCD generally fail to take into account the fundamental role of habits in the formation of chunked action-sequences, i.e., relatively invariant, rapidly deployed sequences of single action-units (Ostlund et al., 2009; Dezfouli and Balleine, 2013). Action sequences, like habits, are mainly dependent on the sensorimotor striatum, with each action unit being triggered by the antecedent action rather than by environmental stimuli. Additionally, in action sequences, assessments of individual action-units and of inter-action states are bypassed (Dezfouli and Balleine, 2013). This could be, to some degree, the opposite of what is frequently described by OCD patients, namely the failure to develop adaptive and automatic action-sequences, performing them with effort, in constant doubt as to their correct execution and hampered by continuous state re-evaluation (Reuther et al., 2013). In fact, in a rat model of pharmacologically-induced compulsivity, evidence was presented that the inability to change between two actions is related to the occurrence of an abnormal phasic dip in VTA dopamine neuron burst firing at the completion of a compulsive-like action sequence, leading to the recurrent activation of the same action, rather than the expected transition to a new action sequence (Joel and Doljansky, 2003). It is interesting, in this respect, that OCD and OCS are particularly frequent in movement disorders that also disrupt the normal chunking of action sequences, such as chorea, Parkinsonism or primary dystonia (Tremblay et al., 2010; Barahona-Corrêa et al., 2011).

The above-mentioned failure to develop adaptive and automatic action-sequences could be interpreted in the context of maladaptive goal-directed uncertainty monitoring, leading to repetitive behaviors, such as checking, in order to reduce uncertainty. This possibility is consistent with the evolving evidence on the role of the STN in the regulation of behavior, and more specifically in the neurobiology and psychopathology of OCD. As mentioned previously, the STN has been one of the targets for DBS in OCD. Linked to frontal cortical areas by the so-called hyperdirect pathway (Nambu et al., 2002), the STN could constitute a potential route of entry, into striatal circuits, of higher-level decision signals for regulation of behavior (Weintraub and Zaghloul, 2013). One of these areas is the OFC, where, in rats, neurons have been shown to encode uncertainty regarding stimulus identity (Kepecs et al., 2008). Since, in OCD patients engaged in repetitive checking bouts, STN neuron spike rates increase prior to the repetition of checking actions (Burbaud et al., 2013), such neural activity changes could reflect uncertainty signals, possibly originating in the OFC, and leading to repetitive goal-directed behaviors, in an attempt to reduce uncertainty.

One other unresolved question regarding the predominance of habitual action strategies in OCD is that such strategies result in highly adaptive action control patterns, allowing the subject to engage in alternate and cognitively demanding tasks, while performing a given well-learned action. This actually seems to be impaired in OCD patients, who not only engage explicit memory-linked medial temporal structures when performing implicit learning tasks (Rauch et al., 2007), but also show impaired sequence learning when faced with parallel, simultaneous explicit and implicit processing demands (Deckersbach et al., 2002). An alternative proposal is that OCD may result from an inability to inhibit the motivation to perform a compulsion, rather than simply from a bias in action control toward habitual behavior (Hinds et al., 2012). Repetition of compulsive behaviors would thus result from a primary overvaluation of action outcomes, with over-activity of the goal-directed action control system that is, by definition, sensitive to outcome values. In an example conceived according to this model, environmental danger-signaling cues could activate the motivation to engage in stereotyped, safety behaviors such as washing or checking, but performance of these actions would fail to effectively terminate the motivational state that elicited them, thus prompting repetition of the behaviors (Woody and Szechtman, 2011; Hinds et al., 2012).

## From Neurobiology to Phenomenology

It is intuitive that the biological understanding of medical disorders should progress from semiology to etiology, rather than the opposite. For most medical disorders, understanding the physiology of signs and symptoms paved the way to the formulation of pathophysiological models and ultimately to the discovery of the disorder’s primary causation. Yet, in the case of OCD, it is clear that the evolution in our understanding of the disorder’s pathophysiology has had a profound impact on our conceptualization of the disorder’s phenomenology. Indeed we may say that the gradual dislocation of the main focus of pathophysiological models of OCD from cortical to subcortical structures has been paralleled by a metamorphosis of the conceptualisation of OCD phenomenology, whereby the initial emphasis laid on the control of explicit thought processes, thought contents, and thought-evoked anxiety, has been replaced by concepts rooted in behavioral neuroscience and animal models of action control. Contemporary literature on OCD is dominated by new phenomenological concepts, and even a new semantics for OCD-related concepts, now seen as characterized by a loss of inhibitory control over impulsive stimuli-response associations, by excessive harm-avoidance, or by a failure to flexibly shift between alternative action-sequences in response to changing environmental demands. Similarly, new insights into the role of dopamine and reward circuits in OCD led researchers in the field to emphasize the phenomenological analogies between addiction disorders and OCD, regarded here as a form of behavioral addiction, a disorder combining impulse control dysfunction with abnormal reward processing mechanisms. These phenomenological metamorphoses found echo in the very nosology of OCD, which in DSM-V was emancipated from anxiety disorders and raised to a new statute as an autonomous spectrum of disorders of behavior control.

In very recent years, the phenomenological analogy between habitual action control and compulsive behavior brought yet another renewal of the semantics and the phenomenology of OCD. The idea that OCD patients may be predisposed to rely excessively on habitual rather than on goal-directed behavioral strategies now faces the field with a host of new concepts stemming from animal experimentation. To some degree, there seems to be a tendency to reduce the psychopathology of OCD to a deregulation of automatic behavior and pathological streams of non-functional stimuli-response associations, downplaying the role of cognition in the process. At the extreme of this evolution from a cognitivist toward a behavioral control centered conceptualisation of OCD, obsessions are eventually seen as secondary, retrospective cognitive constructions developed by the subject to assimilate these intrusive behavioral programs back into the self (Gillan et al., 2011). Compulsions, on the contrary, gradually emerge as the new protagonist in OCD phenomenology, either as merely perseverative phenomena, as stereotyped behaviors pathologically invested with reward value, or as abnormally resilient habitual action-sequences.

## Conclusion

Science has come a long way in understanding OCD. It has been a unique process, marked by dialectic interplay between the progress in neurobiological models of OCD and an increasingly sophisticated phenomenological characterization of this complex syndrome. Refined structural and functional imaging of the brain and technological progress in animal experimental modeling of OCD have opened the way to groundbreaking insights into the role of cortical and subcortical structures in the disorder’s pathophysiology, and revolutionized our perception and interpretation of obsessive-compulsive phenomena. Yet, in spite of much progress, we still lack an explanation for many of the disorder’s manifestations, and not least its extraordinary clinical variability. Extrapolations from animal models, themselves extrapolations of human phenomenology, while carrying extraordinary promise to further understanding of the disorder, necessarily ignore many complex aspects of human psychopathology. Care is thus needed to carefully cross-validate human-animal-human extrapolations, as well as analogies between psychopathology and constructs from experimental psychology and behavioral neuroscience literature, while avoiding impoverishing an extremely rich legacy of psychopathological detail and subtlety. In common with other psychiatric disorders such as bipolar disorder or schizophrenia, neuroimaging studies in OCD are often contradictory and difficult to interpret. This is due not only to phenotypic variability across subjects, but also to the far from clear meaning of volumetric and BOLD-signal differences between clinical subjects and healthy controls. Genetic studies have likewise failed to provide a much anticipated breakthrough in understanding and treating OCD and other mental disorders, probably because genetic factors lie too far upstream in the complex flow of factors and events that ultimately lead to the expression of mental illness in a given individual. A renewed interest for psychopathological refinement can certainly contribute toward the advancement of knowledge on the neurobiology of mental disorders in general, beyond explanation of each syndrome’s gross features and toward tackling of more subtle manifestations and of subtypes of each disorder. Nevertheless, the future also calls for more detailed studies using, for example, genetic animal models, improved neuroimaging technology, *in vivo* neuronal recordings and non-invasive neuromodulation in humans, in order to more fully elucidate the mechanisms that may underlie the details of OCD psychopathology.

### Conflict of Interest Statement

The Reviewer Sérgio Saraiva declares that, despite being affiliated to the same institution as the author Pedro Castro-Rodrigues, the review process was handled objectively and no conflict of interest exists. The other authors declare that the research was conducted in the absence of any commercial or financial relationships that could be construed as a potential conflict of interest.
